# SUVmax on ^18^F‐FDG PET/CT and Histopathological Necrosis in Osteosarcoma and Ewing Sarcoma

**DOI:** 10.1155/bmri/4420962

**Published:** 2026-04-09

**Authors:** Şule Çalışkan Kamış, Metin Çil, Emel Koçyiğit Deveci, Zeynel Abidin Taş, Begül Yağcı

**Affiliations:** ^1^ Department of Pediatric Hematology and Oncology, Adana City Training and Research Hospital, University of Health Sciences, Adana, Turkey, akdeniz.edu.tr; ^2^ Department of Nuclear Medicine, Adana City Training and Research Hospital, University of Health Sciences, Adana, Turkey, akdeniz.edu.tr; ^3^ Department of Medical Pathology, Adana City Training and Research Hospital, University of Health Sciences, Adana, Turkey, akdeniz.edu.tr

**Keywords:** ^18^F-FDG PET/CT, Ewing sarcoma, osteosarcoma, SUVmax, tumor necrosis percentage

## Abstract

**Purpose:**

This study is aimed at evaluating the relationship between preoperative ^18^F‐fluorodeoxyglucose positron emission tomography/computed tomography (^18^F‐FDG PET/CT) findings and histopathological necrosis rates following chemotherapy in patients diagnosed with osteosarcoma (OST) and Ewing sarcoma (EWS).

**Methods:**

Patients diagnosed with OST and EWS between 2017 and 2023 were retrospectively analyzed. Data recorded included preoperative ^18^F‐FDG PET/CT findings, demographic characteristics, histopathological diagnosis, maximum standardized uptake value at diagnosis (SUVmax1), maximum standardized uptake value after neoadjuvant chemotherapy (SUVmax2), the SUVmax change ratio (SUVmax2/SUVmax1 = SCR), and the percentage of tumor necrosis in resected specimens.

**Results:**

A total of 49 patients (33 OST and 16 EWS) were included, consisting of 22 females (44.9%) and 27 males (55.1%) with a median age of 12 years (range: 4–20). Median SUVmax1, SUVmax2, and SCR values were 6.8 (0–22.5), 2.5 (0–9.42), and 0.38 (0–1.44), respectively. The median tumor necrosis percentage was 20% (range: 0–100). Stratification according to SUVmax2 (< 2.5 vs. ≥ 2.5) revealed no significant difference in necrosis percentage (*p* = 0.234). No significant correlation was observed between SCR and necrosis percentage (*p* = 0.102). However, a significant inverse correlation was found between SUVmax2 and necrosis percentage in the overall cohort (*p* = 0.040, *r* = –0.295), which was more pronounced in OST patients (*p* = 0.014, *r* = –0.426).

**Conclusion:**

^18^F‐FDG PET/CT is a valuable imaging modality for predicting histopathological response in solid tumors. Consistent with adult studies, our findings demonstrate that post‐NACT FDG uptake is inversely correlated with tumor necrosis percentage, particularly in pediatric OST patients. These results highlight the potential of ^18^F‐FDG PET/CT as a noninvasive prognostic tool to assist in early identification of poor responders.

## 1. Introduction

Osteosarcoma (OST) is one of the most common primary bone tumors in children, with an annual incidence of approximately 4.4 cases per million children [1, 2]. OST typically arises in the metaphyseal region of long bones. Two‐thirds of tumors occur in the distal femur, making it the most frequent site, followed by the proximal tibia. The proximal humerus accounts for about 10% of cases [3]. The most common presenting symptom is bone pain, which is often severe enough to awaken the patient from sleep. Less frequently, patients may present with pathological fractures or sudden worsening of symptoms. At the time of diagnosis, metastases are detected in nearly 20% of patients. More than 85% of metastatic lesions occur in the lungs, whereas the second most frequent site is distant bones [4]. The presence of metastasis significantly reduces the 5‐year survival rate [5].

Ewing sarcoma (EWS) is the second most common bone tumor in children after OST [6]. It belongs to the group of malignant small round cell tumors [7]. The incidence is approximately three cases per million children annually, and the diagnosis is most often made during adolescence or early adulthood [8, 9]. About 85% of cases originate in the bone, most commonly affecting the pelvis, long bones, and chest wall. Soft tissue EWS is observed in approximately 15% of cases. Nearly 75% of patients present with localized disease, whereas the lungs are the most frequent site of metastasis in advanced cases [10]. At the molecular level, the t(11; 22)(q24; q12) translocation is present in about 90%–95% of patients and serves as a diagnostic hallmark, whereas the t(21; 22)(q22; q12) translocation is observed in 5%–10% of cases [11].

^18^F‐fluorodeoxyglucose positron emission tomography/computed tomography (^18^F‐FDG PET/CT) is a widely used imaging modality in oncology, based on the detection of increased metabolic activity in tumor tissue. The maximum standardized uptake value (SUVmax) is a well‐established parameter used to noninvasively assess tumor biology [12, 13].

In OST and EWS, ^18^F‐FDG PET/CT provides clinically relevant information beyond conventional anatomical imaging. At diagnosis, it supports initial staging by depicting metabolically active primary and metastatic lesions, can aid in detecting occult distant disease, and may contribute to risk stratification [14].

During treatment, changes in tumor glucose metabolism on interim or postneoadjuvant PET/CT have been investigated as early biomarkers of response and prognosis [15, 16]. In particular, residual F‐18 fluorodeoxyglucose (FDG) uptake after neoadjuvant chemotherapy has been associated with histopathological response and survival outcomes in OST [15], whereas evidence in EWS is more heterogeneous, potentially reflecting biological and metabolic variability [16]. Therefore, metabolic parameters such as SUVmax before and after neoadjuvant chemotherapy may complement histopathological necrosis by offering a noninvasive, early indicator of poor response that could inform individualized treatment strategies [15, 16].

Recent studies have suggested that metabolic activity measured by ^18^F‐FDG PET/CT may be influenced by patient‐related factors such as sex, body mass index, and body composition, which can affect both SUV quantification and metabolic interpretation as well as clinical outcome [15–17]. Emerging evidence indicates that sex‐related biological differences and nutritional status may modulate tumor metabolism and chemotherapy sensitivity, highlighting the importance of careful interpretation of metabolic imaging parameters in children and adolescents [15–17]. Incorporating such factors into the evaluation of PET/CT findings may further refine risk stratification and prognostic assessment in pediatric bone sarcomas [12, 13].

In this study, we aim to evaluate the relationship between ^18^F‐FDG PET/CT findings and histopathological necrosis rates following neoadjuvant chemotherapy in pediatric patients diagnosed with OST and EWS.

## 2. Material And Methods

### 2.1. Study Population

This study was designed as a single‐center retrospective cohort study. Between 2017 and 2023, a total of 103 pediatric patients diagnosed with OST or EWS and treated at the Pediatric Oncology Clinic of the University of Health Sciences, Adana City Training and Research Hospital were retrospectively reviewed. Of these, 49 patients (33 OST and 16 EWS) with available preoperative ^18^F‐FDG PET/CT results and documented histopathological tumor necrosis percentage were included in the analysis.

A total of 54 patients were excluded due to incomplete data: 37 with EWS (9 lacking PET/CT results and 28 lacking necrosis percentage) and 17 with OST (5 lacking PET/CT results and 12 lacking necrosis percentage).

For each included patient, demographic characteristics (age and gender), histopathological diagnosis, maximum standardized uptake value (SUVmax1) of the primary tumor at diagnosis, SUVmax after completion of neoadjuvant chemotherapy (SUVmax2), SUVmax change ratio (SUVmax2/SUVmax1 = SCR), and percentage of tumor necrosis in the resected specimen were recorded. Histological response was classified as *good* when necrosis was ≥ 90% and *poor* when < 90%, in line with commonly used histological response criteria in OST treated with neoadjuvant protocols [3].

### 2.2. Chemotherapy Protocol

Neoadjuvant chemotherapy regimens were administered according to institutional pediatric oncology protocols. Patients with OST received multiagent chemotherapy including high‐dose methotrexate, doxorubicin, and cisplatin, whereas patients with EWS were treated with alternating cycles of vincristine, doxorubicin, cyclophosphamide, and ifosfamide/etoposide. Chemotherapy was administered prior to surgical resection, followed by adjuvant therapy according to histopathological response and disease stage.

### 2.3. PET/CT Imaging Protocol

All imaging was performed using a PET/CT system (Biograph mCT, Siemens Healthineers, Erlangen, Germany) under standard whole‐body acquisition protocols. Patients received an intravenous injection of 5.7 mCi (210 MBq) FDG. Prior to tracer administration, blood glucose levels were measured and confirmed to be within the acceptable range. After injection, patients rested in a quiet, dimly lit room without speaking or chewing to minimize physiologic muscle uptake.

Approximately 60 min postinjection, a whole‐body PET/CT scan was performed. A low‐dose CT scan was acquired for attenuation correction and anatomical localization, followed by PET imaging from the skull base to the mid‐thigh (or whole body if clinically indicated). Intravenous contrast material was not routinely administered during CT acquisition, as CT was primarily used for attenuation correction and anatomical localization. PET images were reconstructed with CT‐based attenuation correction and evaluated in transaxial, coronal, and sagittal planes, in addition to maximum intensity projection (MIP) images.

All PET/CT scans were reviewed by an experienced nuclear medicine physician using a standardized institutional protocol. SUVmax measurements were obtained by placing volumes of interest over the most metabolically active portion of the primary tumor on attenuation‐corrected images. To minimize measurement variability, the same reconstruction parameters and acquisition protocol were applied for baseline and postneoadjuvant studies.

All SUV measurements were normalized to body weight, in accordance with standard clinical PET/CT practice. SUVmax was calculated as the highest voxel value within the primary tumor volume of interest on attenuation‐corrected images.

### 2.4. Histopathological Evaluation

Histopathological evaluation of tumor necrosis was performed on surgical resection specimens by an experienced musculoskeletal pathologist. Assessment was based on routine hematoxylin and eosin–stained sections. All available representative sections from the resected tumor specimens were systematically examined for necrosis assessment. The percentage of tumor necrosis was determined by evaluating the entire tumor bed rather than a predefined fixed number of microscopic fields and calculating the proportion of necrotic tissue relative to viable tumor. A hotspot‐based approach was not used; instead, evaluation was performed systematically across representative viable and necrotic areas. Histological response was categorized using a predefined grading scheme, with ≥ 90% necrosis considered a good response. All assessments were conducted using conventional light microscopy (Leica DM2000 LED, Leica Microsystems CMS GmbH, Wetzlar, Germany), and no digital image analysis software was employed. Pathological diagnosis was established based on histomorphology and supported by immunohistochemical analysis where appropriate; molecular analysis was not performed.

### 2.5. Ethics Statement

This study was conducted in accordance with the principles of the Declaration of Helsinki. Ethical approval was obtained from the Adana City Training and Research Hospital Clinical Research Ethics Committee (Decision No. 1744, dated January 27, 2022). As this was a retrospective study, additional informed consent was not required.

### 2.6. Statistical Analysis

Statistical analyses were conducted using IBM SPSS Statistics Version 26.0 (IBM Corp., Armonk, New York, United States). Demographic and clinical characteristics were summarized using descriptive statistics: categorical variables as frequencies and percentages, and continuous variables as mean ± standard deviation or median with range, as appropriate.

SUVmax1, SUVmax2, and SCR values were analyzed in relation to histopathological response status (good vs. poor responders). Sample size estimation was not performed a priori due to the retrospective design and the rarity of pediatric bone sarcomas. The study population represents all eligible patients treated at our institution during the study period with available paired PET/CT data and documented histopathological necrosis percentage. We acknowledge that the modest sample size—particularly for the EWS subgroup—may limit statistical power for subgroup analyses.

Normality of continuous variables was assessed using the Shapiro–Wilk test. Correlations between SUVmax2 and histopathological necrosis percentage were primarily evaluated using Spearman′s rank correlation coefficient due to the bounded nature of necrosis percentage and potential deviations from normality. Pearson correlation analysis was additionally performed for comparison. Group comparisons were conducted to explore differences between good and poor histological responders rather than to formally assess discriminatory performance.

A two‐sided *p* value ≤ 0.05 was considered statistically significant for all analyses.

## 3. Results

The study included a total of 49 patients, of whom 33 (67.3%) were diagnosed with OST and 16 (32.7%) with EWS. There were 22 females (44.9%) and 27 males (55.1%), yielding a male‐to‐female ratio of 1.22. The median age at diagnosis was 12 years (range: 4–20 years). The largest age group was 10–15 years (44.9%). Specifically, 2 patients (4.1%) were under 5 years old, 11 (22.4%) were between 5–10 years, 22 (44.9%) were between 10–15 years, 13 (26.5%) were between 15–20 years, and 1 patient (2%) was older than 20 years. The demographic and clinical characteristics of the patients are summarized in Table 1.

**Table 1:** Demographic characteristics and clinical findings of the patients.

Variable	Osteosarcoma	Ewing sarcoma	All patients
Median age, years (range)	12 (4–20)	13.5 (4–19)	12 (4–20)
Gender—Female, *n*	16	6	22
Gender—Male, *n*	17	10	27
Diagnosis, *n*	33	16	49
SUVmax1, median (range)	7.0 (0–22.5)	6.54 (2–10.17)	6.8 (0–22.5)
SUVmax2, median (range)	2.65 (0–9.42)	2.26 (0–9.3)	2.5 (0–9.42)
SCR, median (range)	0.38 (0–1.20)	0.37 (0–1.44)	0.38 (0–1.44)
Necrosis %, median (range)	30 (0–100)	17.5 (0–100)	20 (0–100)

The overall median SUVmax1 was 6.8 (range: 0–22.5), SUVmax2 was 2.5 (range: 0–9.42), and the SCR value was 0.38 (range: 0–1.44). The median tumor necrosis percentage was 20% (range: 0–100). A subgroup analysis showed that the median necrosis percentage was higher in OST (30%) compared with EWS (17.5%). Subgroup analyses were performed to explore potential differences between OST and EWS patients and were considered exploratory in nature.

Histopathological evaluation revealed a good response to chemotherapy (≥ 90% necrosis) in 6 patients (12.2%) and a poor response (< 90% necrosis) in 43 patients (87.8%). At the time of analysis, 16 patients (32.7%) were in disease‐free remission, 13 (26.5%) were alive with disease and receiving chemotherapy, 7 (14.3%) were lost to follow‐up, and 13 (26.5%) had died. Relapse occurred in 29 patients (59.2%), whereas 20 patients (40.8%) remained relapse‐free.

When patients were divided into two groups according to SUVmax2 (< 2.5 vs. ≥ 2.5), there was no statistically significant difference in necrosis percentage between the groups (*p* = 0.234). Similarly, no significant correlation was found between the SCR value and necrosis percentage (*p* = 0.102). However, a significant inverse correlation was observed between SUVmax2 and necrosis percentage in the overall cohort (*p* = 0.040, *r* = –0.295). This association was stronger in the subgroup of OST patients, where SUVmax2 showed a significant inverse correlation with necrosis percentage (*p* = 0.014, *r* = –0.426).

## 4. Discussion

^18^F‐FDG PET/CT is widely used in musculoskeletal system tumors for initial staging, detection of distant metastases or early recurrences, restaging, monitoring treatment response, and guiding subsequent therapeutic strategies [13].

In our study, we evaluated the relationship between PET/CT metabolic parameters and histopathological tumor necrosis in pediatric patients with OST and EWS. The main finding was that a significant inverse correlation between post‐NACT SUVmax (SUVmax2) and tumor necrosis percentage was observed in the overall cohort, which was more pronounced in the OST subgroup (Figure 1). This means that patients with higher residual FDG uptake after chemotherapy tended to show lower histological necrosis, suggesting poor treatment response. Clinically, this highlights the prognostic value of SUVmax2 in early identification of patients who may require alternative or intensified treatment regimens.

Figure 1Representative ^18^F‐FDG PET/CT findings before and after neoadjuvant chemotherapy in pediatric osteosarcoma. (a) Baseline ^18^F‐FDG PET/CT images obtained at diagnosis demonstrating intense FDG uptake in the primary distal femoral lesion on axial fusion, coronal maximum intensity projection (MIP), coronal CT, and sagittal CT images. (b) Postneoadjuvant chemotherapy PET/CT images showing decreased but persistent residual metabolic activity in the same lesion. The reduction in metabolic activity after treatment, with residual FDG uptake, corresponds to incomplete histopathological tumor necrosis, supporting the observed inverse correlation between posttreatment SUVmax and tumor necrosis percentage.

**Figure:**
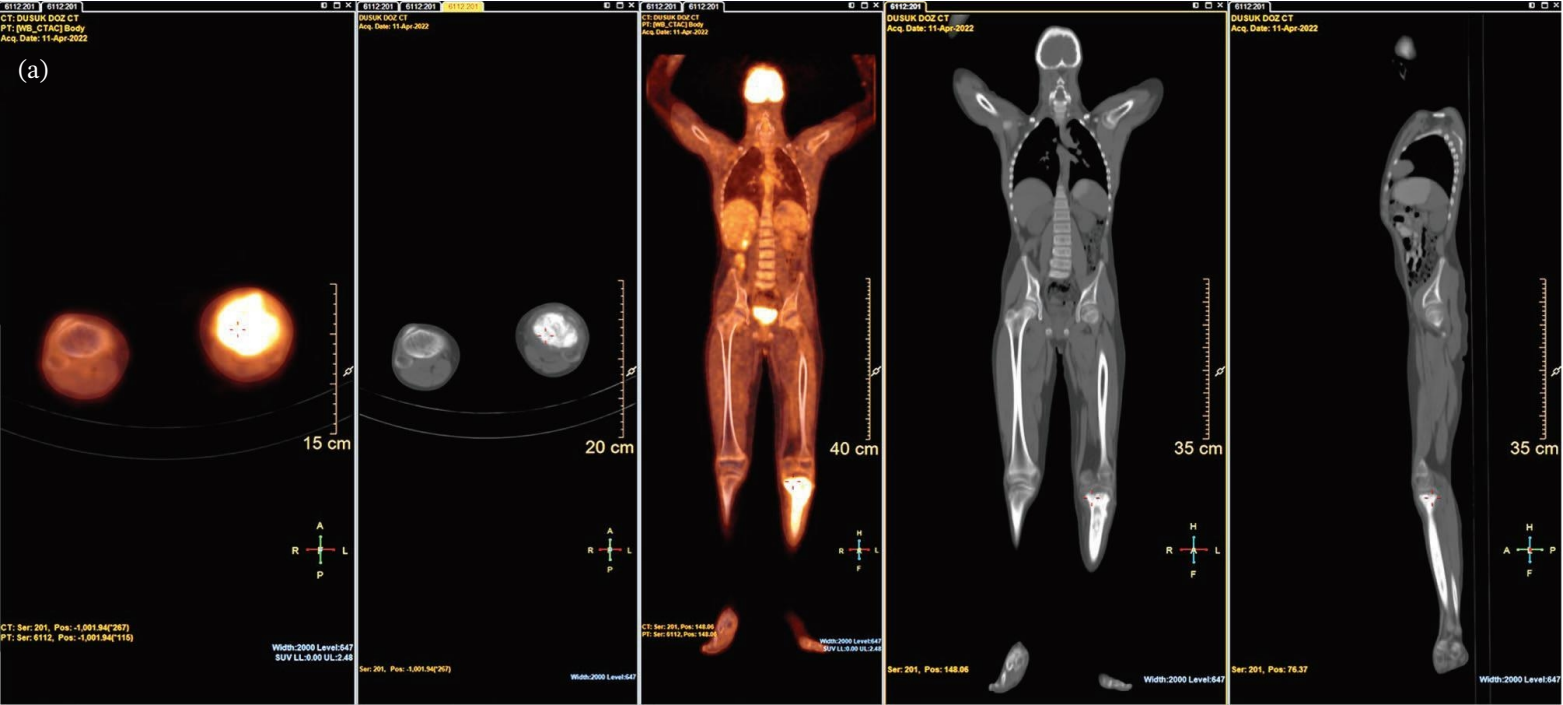
(a)

**Figure:**
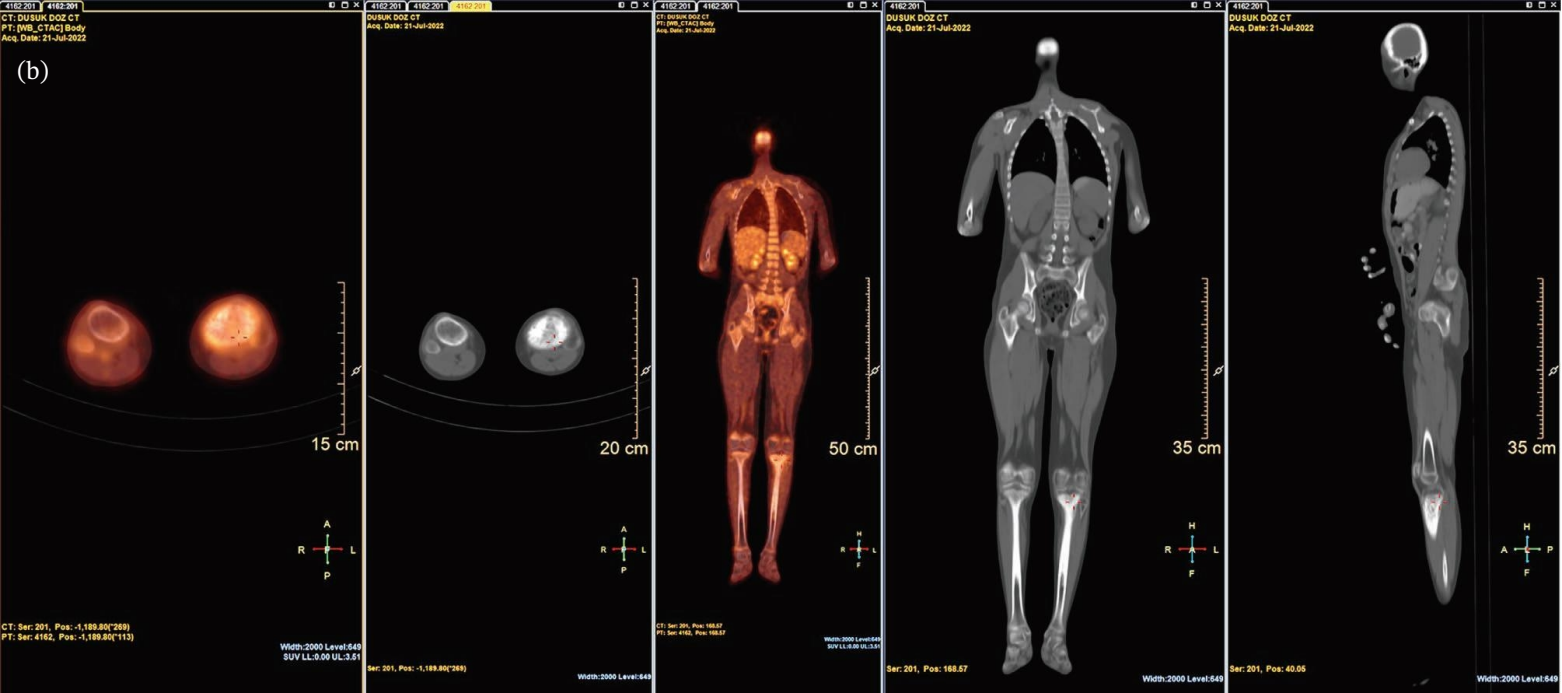
(b)

The observed inverse association between postneoadjuvant SUVmax and histopathological necrosis suggests that metabolic response on ^18^F‐FDG PET/CT may serve not only as a surrogate marker of chemotherapy efficacy but also as a prognostic indicator. Early identification of patients with persistently high metabolic activity despite neoadjuvant therapy may allow consideration of alternative or intensified treatment strategies.

This is particularly relevant in pediatric OST, where histopathological response remains one of the strongest predictors of outcome. Expanding evidence from pediatric studies supports the integration of metabolic imaging into response‐adapted treatment algorithms [18, 19].

Our findings are consistent with previous studies. Cheon et al. demonstrated that ^18^F‐FDG PET/CT could predict histological response to NACT in OST [14]. Similarly, other publications reported a correlation between reduced glucose metabolism after chemotherapy and favorable histological responses in primary bone sarcomas [18, 19]. In pediatric OST patients, post‐NACT PET/CT has been shown to predict poor histological response, which is in line with our results [20]. Furthermore, metabolic parameters derived from PET/CT at staging, interim, and posttreatment have been associated with necrosis percentage, progression‐free survival, and overall survival, underscoring their prognostic significance [21]. Hawkins et al. also reported that baseline SUVmax independently predicted survival outcomes beyond histological grading, supporting the prognostic role of PET/CT [22].

Interestingly, in our study, no significant association was found between SUVmax values and histopathological necrosis in EWS patients. This may be due to biological differences between OST and EWS, heterogeneity in tumor metabolism, or limited sample size. Larger multi‐institutional studies are required to clarify the predictive value of PET/CT in EWS.

Overall, our results confirm the role of ^18^F‐FDG PET/CT as a noninvasive imaging tool not only in staging but also in predicting chemotherapy response, particularly in OST.

This study has limitations. First, its retrospective single‐center design and the modest sample size, particularly in the EWS subgroup, may have reduced the power to detect associations and limited generalizability. Second, treatment regimens may have varied across patients, which could have influenced metabolic response and necrosis rates. Third, response assessment relied on histopathological necrosis and did not incorporate standardized radiological response criteria such as RECIST alongside metabolic parameters. Future multicenter studies with larger, more homogeneous pediatric cohorts are warranted to validate the prognostic utility of interim and postneoadjuvant ^18^F‐FDG PET/CT in bone sarcomas.

## 5. Conclusion

Numerous studies have explored the association between imaging modalities and histopathological response to chemotherapy in solid tumors. In adult OST, ^18^F‐FDG PET/CT has been shown to predict the extent of tumor necrosis. Consistent with these findings, our study demonstrated that postneoadjuvant chemotherapy FDG uptake (SUVmax2) was inversely correlated with tumor necrosis percentage, particularly in pediatric OST. These results highlight the potential of ^18^F‐FDG PET/CT as a valuable noninvasive imaging biomarker for early identification of poor responders and for guiding individualized treatment strategies in pediatric bone sarcomas. Future prospective multicenter studies are warranted to validate these findings and to further refine risk stratification using integrated metabolic and clinical parameters.

## Author Contributions

Ş.Ç.K.: conception, study design, data collection and/or processing, manuscript writing, and final approval. M.Ç.: critical revision, editing, and supervision. E.K.D.: nuclear medicine data collection, PET/CT image evaluation, and interpretation. Z.A.T.: pathological evaluation, histopathological data analysis, and interpretation. B.Y.: supervision, design, analysis, interpretation, and final approval.

## Funding

No funding was received for this manuscript.

## Disclosure

This study was previously presented as a poster (P‐34) at the XXII National Pediatric Cancer Congress, March 8–12, 2023, Antalya, Turkey.

## Ethics Statement

The study was approved by the Adana City Training and Research Hospital Clinical Research Ethics Committee (Decision No. 1744, January 27, 2022) and conducted in accordance with the principles of the Declaration of Helsinki.

## Consent

The requirement for informed consent was waived by the ethics committee due to the retrospective nature of the study.

## Conflicts of Interest

The authors declare no conflicts of interest.

## Data Availability

The datasets generated and/or analyzed during the current study are not publicly available due to ethical restrictions and participant confidentiality, but are available from the corresponding author upon reasonable request.
